# Simultaneous Spontaneous Bilateral Tension Pneumothorax Post COVID-19 Infection: A Case Study

**DOI:** 10.7759/cureus.25107

**Published:** 2022-05-18

**Authors:** Ciara June, Chad Viscusi, Burke DeLange

**Affiliations:** 1 Medicine, University of Arizona College of Medicine – Tucson, Tucson, USA; 2 Emergency Medicine, University of Arizona College of Medicine – Tucson, Tucson, USA; 3 General and Vascular Surgery, Summit Healthcare Regional Hospital, Show Low, USA

**Keywords:** covid-19 infection, sars-cov-2, covid-19 pneumonia, pneumothorax, tension pneumothorax, spontaneous bilateral pneumothorax

## Abstract

Simultaneous bilateral spontaneous pneumothorax is a rare life-threatening condition that can cause severe respiratory distress, hypoxemia, and death. Spontaneous pneumothorax has been reported as an uncommon but severe complication in patients recovering from COVID-19 pneumonia. Even fewer cases of spontaneous bilateral tension pneumothorax have been reported as a result of infection.

We present a patient with spontaneous bilateral tension pneumothorax 18 days after COVID-19 infection. The patient’s symptoms began with a substernal tearing sensation and pain radiating to the back with dyspnea. Physical exam was significant for oxygen saturation of 75% on room air, tachycardia, and diminished breath sounds bilaterally. Imaging confirmed large bilateral pneumothoraces, and chest tubes were inserted emergently to restore lung volume.

Pneumothorax is predominantly observed in those with severe infection but has been seen with mild symptoms as well. The development of pneumothorax is thought to result from diffuse lung injury that occurred from a cytokine storm during COVID infection. We speculate that our patient developed bulla as a result of infection, and the bulla spontaneously ruptured, inducing the bilateral collapse of the lungs. The mortality of such an event remains unknown, but without proper intervention, mortality increases significantly in these patients.

As we continue to learn about the multitude of sequelae that can result from COVID-19 infection, pneumothorax should be considered in patients with a history of COVID pneumonia that presents with acute onset of dyspnea and chest pain. It is important to quickly recognize such cases as these patients have a narrow time frame for intervention, especially in the event of bilateral tension pneumothorax. Therefore, pneumothorax should be adequately assessed on initial examination, with the possibility of bilateral pneumothoraces in mind, to minimize morbidity and mortality.

## Introduction

Pneumothorax develops when there is an opening between the lung and pleural space usually caused by trauma, iatrogenic lung injuries, or lung disease. A pneumothorax is considered spontaneous when air accumulates in the pleural cavity without direct injury to the lung. It is further stratified into primary and secondary spontaneous pneumothoraces. Primary spontaneous pneumothorax occurs without any known causes to precipitate pneumothorax. Secondary spontaneous pneumothorax is considered when pneumothorax occurs spontaneously in the presence of lung disease [1].

Some cases can present with tension or progress to a tension pneumothorax over time if intervention is delayed. Tension pneumothorax develops when the opening to the pleural space acts as a one-way valve. Air enters the pleural space and becomes trapped, increasing intrapleural pressure and causing a further collapse of the lung. The heart and surrounding vasculature can be compressed by increased pressure, resulting in cardiopulmonary dysfunction and hemodynamic instability. Clinical presentation of pneumothorax includes dyspnea, pleuritic chest pain, tachypnea, tachycardia, absent or diminished breath sounds, and hyperresonance with percussion. Tension pneumothorax has a similar presentation but is usually more dramatic with hypotension, tracheal deviation to the contralateral side, cyanosis, and altered mental status. A chest x-ray may demonstrate a tracheal or mediastinal shift, complete collapse of the ipsilateral lung, or flattening of the diagram [2,3].

Simultaneous spontaneous bilateral tension pneumothorax occurs when both lungs collapse without direct injury and tension clinical findings listed above are present. This can lead to respiratory failure, cardiac arrest, and fatal consequences if left untreated. Therefore, early recognition and immediate intervention are important to minimize adverse outcomes and decrease morbidity and mortality. Diagnosis of pneumothorax can be made through clinical examination at the bedside, but diminished bilateral breath sounds should not be mistaken for normal breath sounds [3]. Confirmation of pneumothorax can be made at the bedside with a portable chest x-ray. In the instance of bilateral pneumothoraces, it is important to consider the presence of tension as tracheal and mediastinum deviation may not be present on physical exams or imaging [4]. We report a case with spontaneous bilateral tension pneumothorax after a recent COVID-19 infection.

## Case presentation

A 54-year-old male presents to the emergency department with acute onset of dyspnea and substernal chest pain radiating to the back. He was diagnosed with COVID-19 pneumonia 18 days prior and required oxygen via a high-flow nasal cannula. He was weaned off the supplemental oxygen four days before presenting to the emergency department. He had no other pre-existing medical conditions and no history of smoking. At the time of onset, he was practicing breathing exercises when a sharp tear was felt in the middle of his chest. The pain radiated to the back, and he began to have dyspnea and tightness in his chest with each breath.

Upon arrival, he was toxic appearing, pale, diaphoretic, and in severe respiratory distress. His trachea was midline with diminished breath sounds bilaterally. The patient was tachycardic and tachypneic; blood pressure was elevated, and oxygen saturation was 75% on room air. Supplemental oxygen was given at 15 L/min through a nonrebreather mask. Heart rate decreased from 135 bpm to 119 bpm; respiratory rate decreased from 34 to 28 breaths per minute, and oxygen saturation increased to 95% with oxygen administration. 

**Figure 1:**
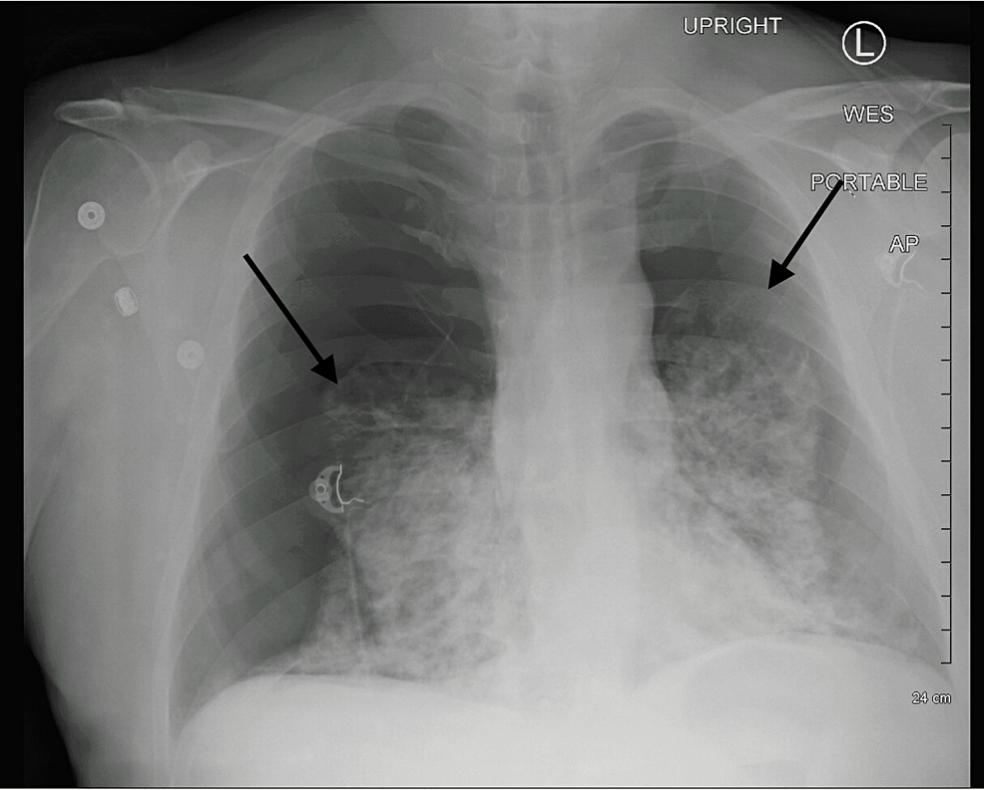
Chest x-ray: spontaneous bilateral pneumothorax with severe airspace disease. The diaphragm is flattened, and the thoracic cage is expanded.

**Figure 2:**
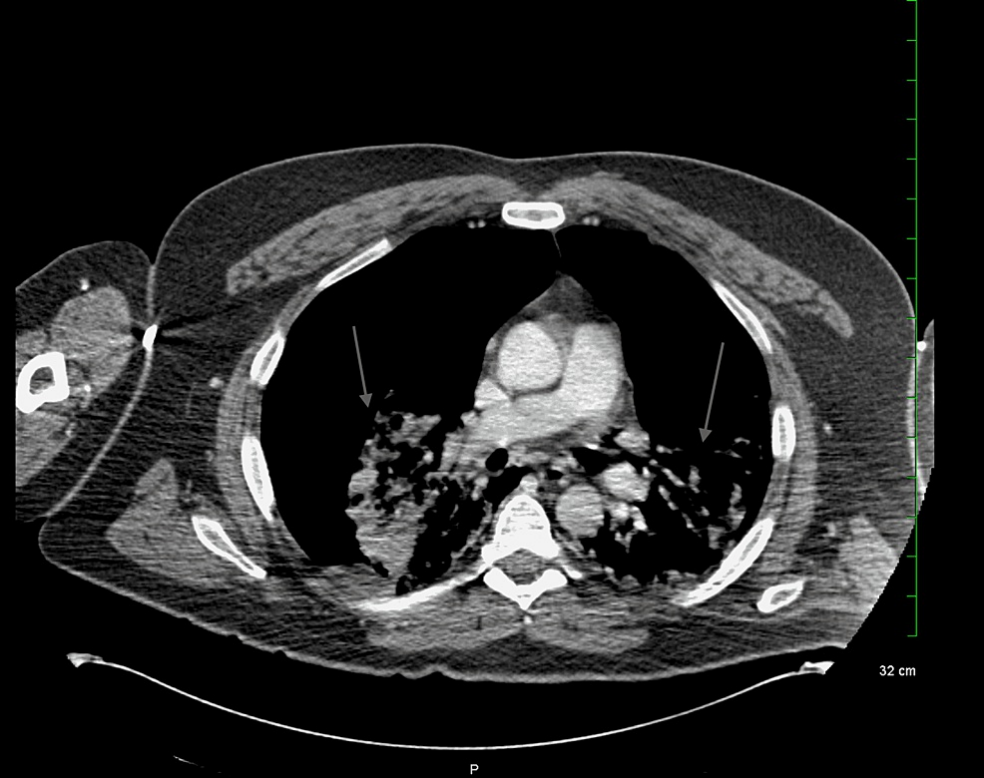
Bilateral pneumothorax (axial view)

A chest x-ray (Figure 1) was taken, and the patient was rushed to radiology for a CT scan (Figure 2) due to concern for aortic dissection. He began to decompensate; hemodynamic instability was noted, but blood pressure was not recorded, and the patient lost consciousness. Intubation was initiated due to acute hypoxic respiratory failure, severe respiratory distress, and hypoventilation.

**Figure 3:**
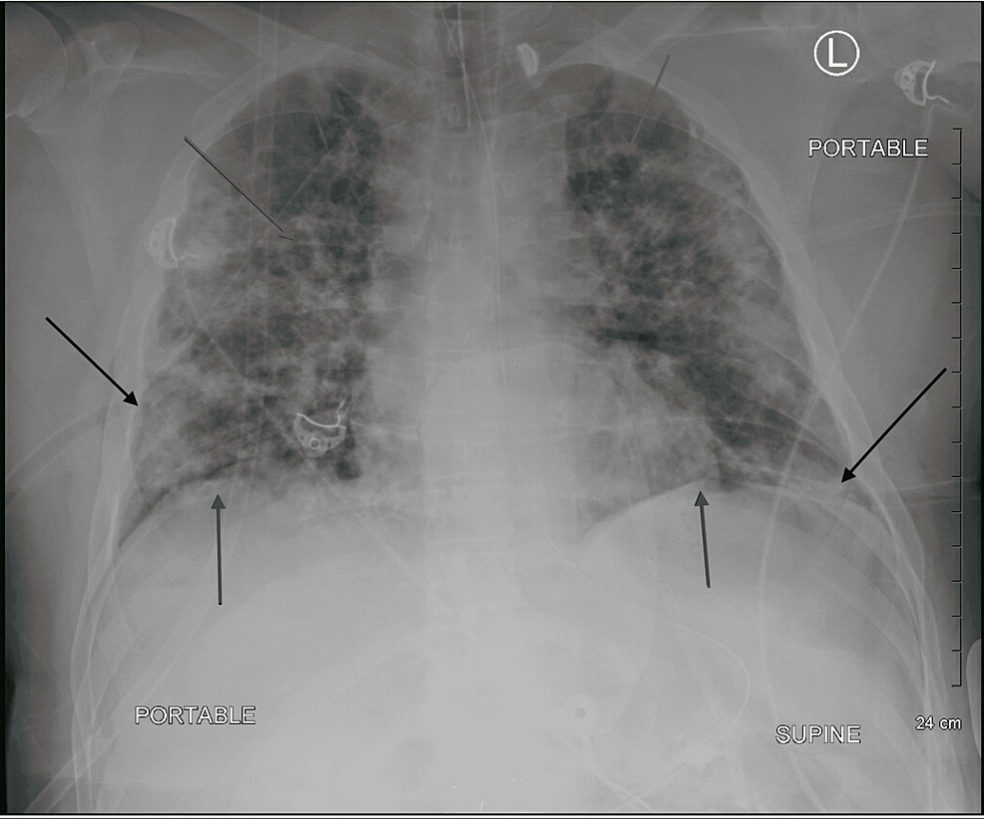
Chest x-ray: bilateral chest tube insertion (black arrows), lung re-expansion, and diaphragm returned to a dome shape (dark gray arrows). Diffuse airspace opacities and cysts are shown in light gray arrows.

**Figure 4:**
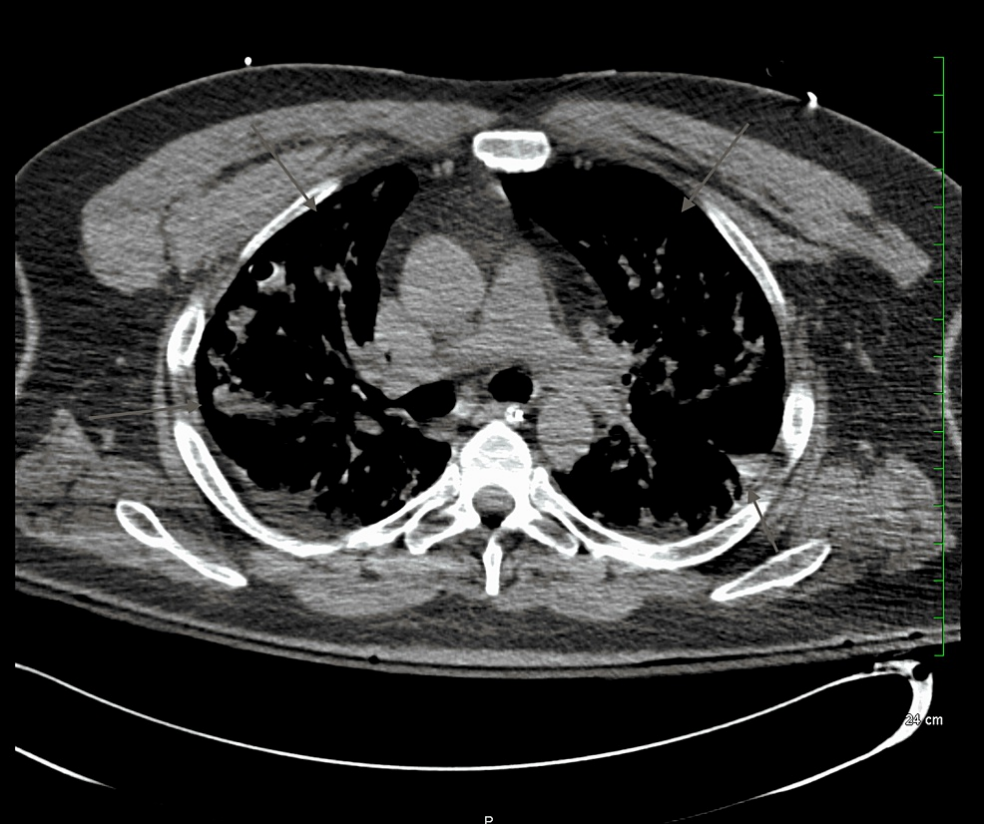
CT scan taken after bilateral chest tube insertion and lung re-expansion (axial view)

Bilateral pneumothorax was noted on imaging with significant lung collapse, and 28F chest tubes were inserted into the right and left pleural spaces at the fifth and seventh intercostal spaces, respectively. Upon insertion, a rush of air was released indicating that tension was present. Oxygen saturations rapidly improved, and a second chest x-ray and CT scan (Figures 3, 4) were obtained to assess the chest tube placement. A large bleb on the left lung was noted during the second CT scan. Bilateral diffuse airspace opacities consistent with COVID pneumonia were shown on imaging.

The patient was transferred to the ICU for monitoring and weaned off the ventilator 24 hours later. Chest tubes were clamped on the fourth day, but there was a persistent right-sided leak, and a chest x-ray showed small residual pneumothorax on the right with subcutaneous emphysema. The left chest tube was removed the following day, and on the sixth day, the right chest tube was removed as the air leak resolved. On the seventh day, the chest x-ray demonstrated improvement in subcutaneous emphysema. The patient was discharged with a scheduled follow-up for a surgical consult for video-assisted thoracoscopic surgery (VATS) procedure and pleurodesis.

## Discussion

The incidence of spontaneous pneumothoraces ranges from 1.4% to 7.6% [4]. Of those cases, approximately 1.3% are simultaneous bilateral spontaneous pneumothoraces [5]. Bilateral pneumothorax classically develops from direct injury to the lung from either trauma, mechanical ventilation, or other iatrogenic causes. When a pneumothorax develops in the absence of these precipitating factors, it is considered spontaneous, as seen in our patient. Spontaneous pneumothorax often occurs in the presence of secondary causes from chronic lung diseases. Chronic obstructive pulmonary disease, cystic fibrosis, tuberculosis, and neoplasms are some of the more commonly reported secondary causes of spontaneous bilateral pneumothorax. Other risk factors of pneumothorax include smoking, male, tall, and thin body habitus [4,6,7].

Spontaneous pneumothorax has been reported as a complication to SARS-CoV-2 infection [8,9]. A majority of the described cases involve unilateral pneumothoraces. To our knowledge, there are only a handful of reports involving spontaneous bilateral pneumothorax as a complication to COVID-19 pneumonia [10,11]. There are even fewer cases involving bilateral pneumothorax with tension. At the time this case study was written, only two other cases of simultaneous spontaneous bilateral tension pneumothorax have been reported after COVID-19 infection [12,13].

Our patient developed spontaneous bilateral tension pneumothorax 18 days after being diagnosed with COVID-19. He had no previous risk factors other than being male. The patient’s symptoms began with a sudden tear in his chest with chest pain that radiated to his back. He was diaphoretic, tachypneic, and had tachycardia, and oxygen saturation was 75% at the time of admission to the emergency department. He demonstrated signs of hemodynamic instability as his symptoms progressed. He lost consciousness and his blood pressure decreased but was not recorded due to the need for urgent intervention; emergent intubation was initiated with decompressive tube thoracostomy.

Upon tube insertion, a rush of air was observed bilaterally, indicating tension was present within the thorax. On this observation, the diagnosis of bilateral tension pneumothorax was made. The lungs are significantly collapsed on the chest x-ray but not fully collapsed as expected with tension pneumothorax in the absence of disease. As a result of COVID-19 pneumonia and diffuse airspace opacities demonstrated on x-ray, this patient’s lungs likely had decreased compliance due to stiffening of the lung parenchyma, decreasing its ability to fully collapse. After lung re-expansion, a bulla on the left apical lung was identified on a CT scan. The bulla may have developed from lung destruction caused by inflammatory proteases released during infection. One bulla possibly ruptured, causing the collapse of the ipsilateral lung, and the force of collapse could have induced collapse in the contralateral lung.

With the global spread and continuation of COVID-19 variants, there is a growing concern surrounding the short-term and long-term consequences it has on the body. The incidence of spontaneous pneumothorax after COVID-19 infection remains unknown but is thought to be around 0.66% according to a retrospective study involving 902 diagnosed COVID-19-infected patients [6]. Consistent with our patient’s presentation, spontaneous pneumothorax has been frequently reported to develop around two to three weeks after COVID-19 infection. The majority of cases are seen in individuals who had a severe illness and acute respiratory distress syndrome (ARDS), but there are some reports of pneumothorax in patients who experienced mild symptoms [6,8,14,15]. As demonstrated in our patient, the residual impact of the disease may persist for weeks after recovery, putting these patients at high risk for morbidity and mortality. Further evaluation of mortality still needs to be done, but according to two studies, pneumothorax in patients with severe illness can increase mortality to as high as 66% to 80% [6,8]. Therefore, it is imperative for clinicians to include spontaneous pneumothorax in the differential diagnosis for patients with a history of COVID-19 infection when presenting with acute chest pain and dyspnea.

## Conclusions

As COVID-19 continues to exist throughout the world, it is important to familiarize ourselves with the varying complications that can precipitate from the infection. Spontaneous pneumothorax is reported as a rare but potentially life-threatening complication that can occur from SARS-CoV-2 infection. Pneumothorax is frequently reported to manifest two or three weeks after infection but has been seen in varying time frames. These patients may still be experiencing residual effects of the diseased lung, such as diffuse airspace infiltrates, resulting in a decreased capacity for adequate ventilation. Therefore, patients may be at greater risk for rapid decompensation and fatal consequences as high mortality has been assessed, but further evaluation still needs to be done. Mortality may be even higher for those who present with spontaneous bilateral tension pneumothorax due to the effect on hemodynamic stability. There is a narrow window before severe deterioration can occur, requiring intubation and resuscitation. Diagnosis and treatment can be completed quickly at the bedside based on physical exam findings and confirmed with a portable chest x-ray. Therefore, prompt recognition and intervention are essential to increasing the chance of survival.
